# Favorable Outcome Twenty-Two Months after Delbet Type Ib Capital Femoral Transepiphyseal Fracture and Posterior Column Acetabular Fracture in a Fifteen-Year-Old Male

**DOI:** 10.1155/2022/1843367

**Published:** 2022-04-21

**Authors:** Saranya A. Sethuraman, Ashlyn S. Morse, Javier Z. Guzman, Robert Cristofaro, David E. Asprinio

**Affiliations:** ^1^Westchester Medical Center, Department of Orthopaedic Surgery, 100 Woods Road, Valhalla, NY 10595, USA; ^2^Mercy Medical Center, Institute for Foot and Ankle Reconstruction, 345 St. Paul Place, Baltimore, MD 21202, USA

## Abstract

**Case:**

A fifteen-year-old male patient sustained a posteriorly dislocated right capital femoral Delbet type Ib epiphyseal fracture-separation and a right acetabular posterior column fracture after a low-energy trip and fall. The capital femoral epiphysis was closed reduced and fixed with cannulated screws on an urgent basis. He underwent acetabular osteosynthesis via a Kocher-Langenbeck approach two days thereafter. Twenty-two months after injury, he was weight-bearing on the right lower extremity without radiologic evidence of avascular necrosis or clinical evidence of pain or functional deficit.

**Conclusion:**

Fracture-separation of the capital femoral epiphysis comprises only 8% of skeletally immature femoral neck fractures in the Delbet and Colonna classification. Prognosis is worse with ipsilateral hip dislocation due to the risk of avascular necrosis from disruption of the medial femoral circumflex artery. Urgent referral to a trauma center and treatment by appropriate specialists enables good long-term results after this uncommon traumatic injury pattern.

## 1. Introduction

Acetabular fractures in skeletally immature patients are relatively rare. In a review of over 7,000 patients in the German Pelvic Trauma Registry, only 153 patients were less than fourteen years old (all older adolescents were excluded as their triradiate cartilage was assumed to be fused), of which only fifteen (9.8%) sustained acetabular fractures [1]. Four had posterior column fractures, and three had posterior wall fractures. Two patients sustained epiphysiolysis of the triradiate cartilage that resulted in premature closure and subsequent acetabular dysplasia. Scuderi and Bronson reported a series of traumatic disruptions of the triradiate cartilage in eleven pediatric patients complicated by premature closure of the triradiate cartilage, leading to acetabular dysplasia and hip subluxation. Younger age was the most significant factor leading to larger growth disturbance, and Salter-Harris V (SH V) fractures were most likely to lead to premature closure while SH I and II fractures had more favorable prognoses [2].

Traumatic hip dislocations are more common than acetabular fractures in the pediatric population and often associated with sports activities or high-energy trauma; a case series of six patients from Turkey in 2014 included five posterior hip dislocations, one of which involved a transepiphyseal fracture [3]. In a 1989 series from the Netherlands, Heeg et al. reported twelve femoral head dislocations associated with acetabular fractures, nine of which were posterior [4]. Seven of the 23 acetabular fractures in that series were posterior column fractures [4].

Delbet and Colonna classified fracture-separation of the capital femoral epiphysis as a rare lesion accounting for just eight percent of femoral neck fractures in skeletally immature patients (Table 1; [5, 6]). Prognosis in these injuries worsens with associated ipsilateral hip dislocation due to the high risk of avascular necrosis of the capital femoral epiphysis from loss of the posteriorly directed blood supply from the medial femoral circumflex artery [6, 7]. Delbet type Ia fractures are those in which the capital epiphysis remains within the acetabulum, while in type Ib fractures, the capital epiphysis is dislocated, with a reported AVN rate after reduction of 74% [8]. The reported incidence of AVN after a Delbet type I injury is 80-100% [9]. Most (75-92%) hip dislocations in adolescents are posterior [10].

These case series spanning the past four decades demonstrate the rarity of each individual injury pattern, and there are no previous case reports of this combination of injuries in a skeletally immature patient. This case report provides valuable insight into treatment options and patient counseling for physicians who may encounter patients with similar injuries in the future.

## 2. Case Presentation

A fifteen-year-and-four-month-old male stumbled late in the evening while running over uneven ground and felt his right leg give away. He was unable to bear weight on the right lower extremity after falling. He was transferred from a referring facility overnight with a shortened, externally rotated right lower extremity and his distal neurovascular exam was intact. Body mass index was 24.4. The patient used albuterol as needed for asthma and seasonal allergies and had an otherwise unremarkable medical history. He reported no prodromal right hip pain.

**Figure 1:**
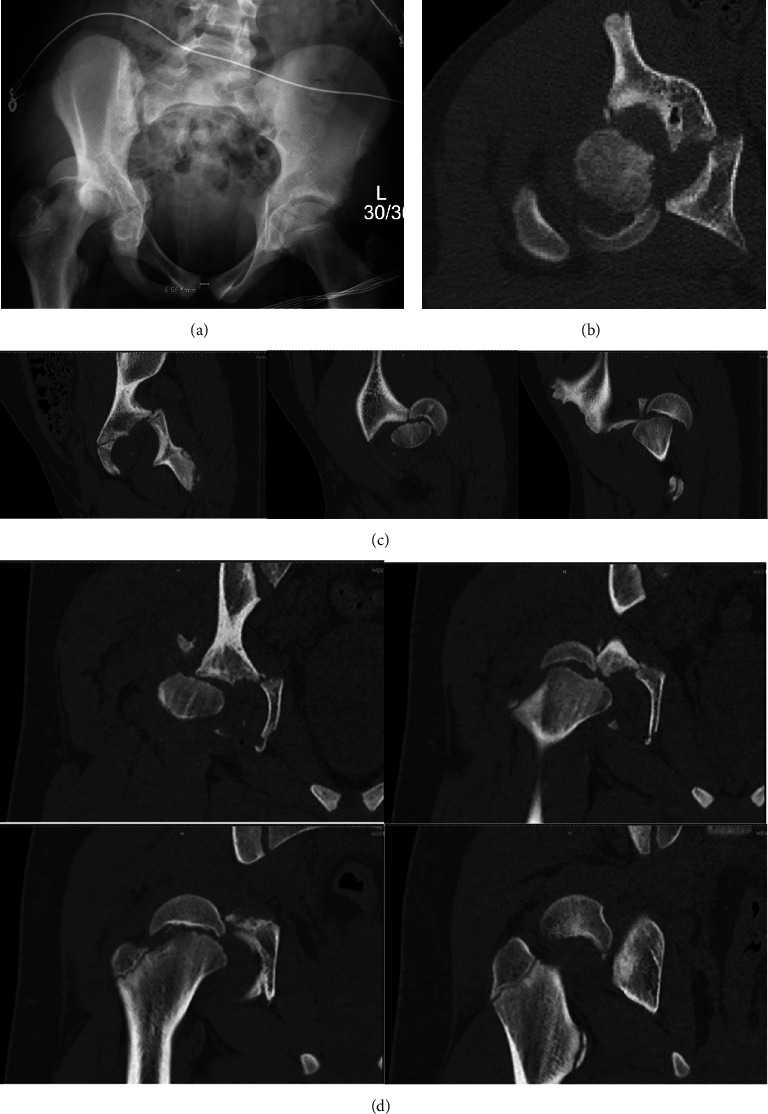
(a) Injury radiographs and selected (b) axial, ((c) 1–3) sagittal, and ((d), 1–4) coronal computed tomography cuts demonstrating displaced right capital femoral epiphysis and posterior fracture-dislocation of right hip with posterior column acetabular fracture.

**Figure 2:**
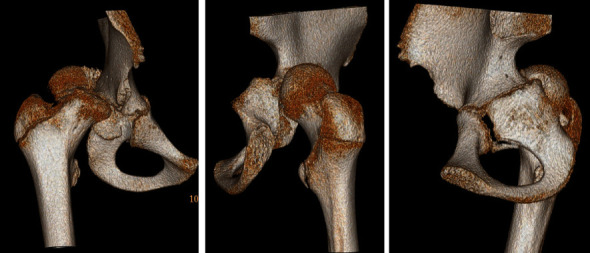
Three-dimensional CT reconstruction images demonstrating the displaced right capital femoral epiphysis and disrupted right acetabular posterior column.

Radiographs and computed tomography (CT) of the pelvis and right femur (Figure 1) showed a posteriorly dislocated Delbet type Ib right capital femoral epiphyseal separation and a fracture of the right acetabulum through the triradiate cartilage, posterior column, and posterior wall. Three-dimensional CT reconstruction images are shown in Figure 2. Closed reduction of the fracture-dislocation was not attempted in the emergency department due to the concern of further disruption of the blood supply to the capital femoral epiphysis.

**Figure 3:**
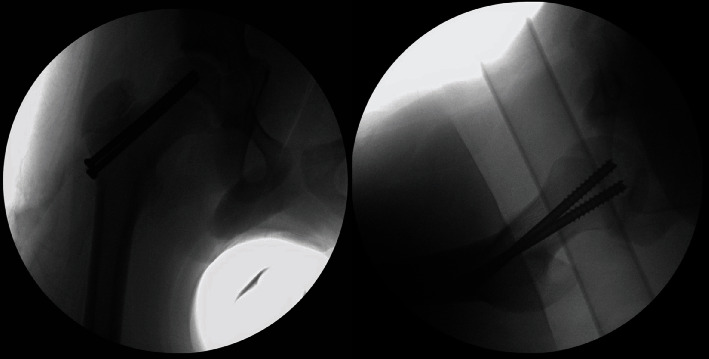
Intraoperative AP and lateral fluoroscopy views demonstrating the reduced right hip joint and fixation of right capital femoral epiphysis.

The patient was taken urgently to the operating room on the day of presentation by a pediatric orthopaedist (R.C.) for closed manipulation and reduction using axial traction. Given the low energy mechanism and resultant posteroinferiorly displaced capital femoral epiphyseal fracture-separation, and the absence of any epiphyseal fragments remaining attached to the distal femoral neck fracture fragment, the displaced physeal fracture-separation was closed reduced with minimal manipulation. The reduced capital epiphysis was percutaneously stabilized with two 6.5 mm cannulated screws (Figure 3). The femoral head was noted to remain posteriorly subluxated due to the posterior column acetabular fracture, prompting placement of a distal femoral skeletal traction pin.

**Figure 4:**
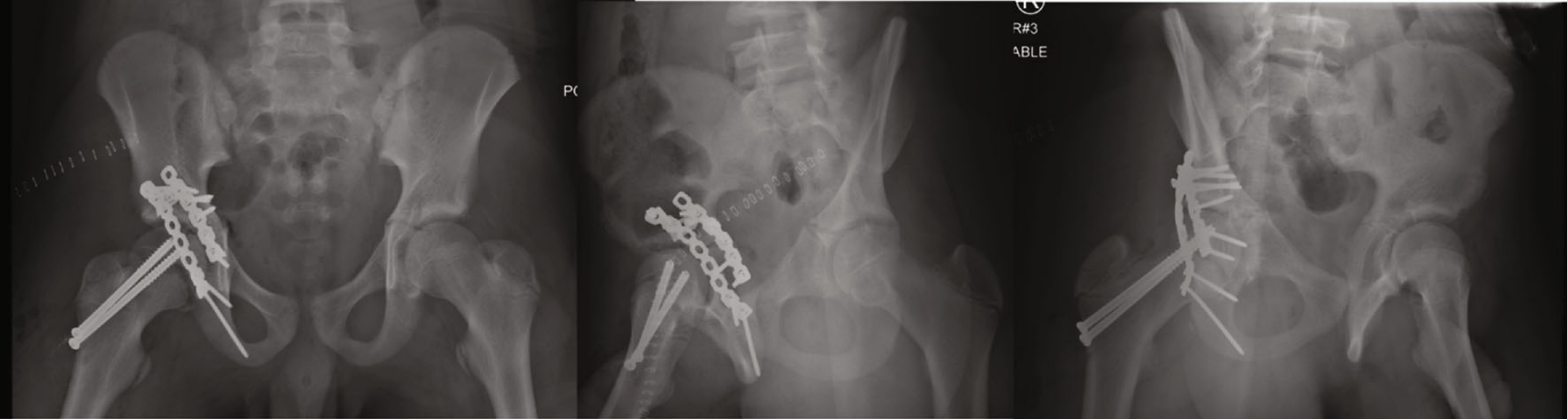
Postoperative AP and Judet pelvis radiographs demonstrating the fixation of right acetabulum and concentric reduction of right femoral head.

The patient then returned to the operating room on hospital day two for open reduction and internal fixation of the right acetabulum (Figure 4) via a Kocher-Langenbeck approach under the direction of an orthopaedic traumatologist (D.E.A.) using a 1-hole spring plate, two 3.5 mm reconstruction plate, and 3.5 mm bicortical position screws. The patient was instructed to be nonweight-bearing on the right lower extremity for eight weeks postoperatively and to follow posterior hip precautions. He was given six weeks of chemoprophylaxis for venous thromboembolism with low molecular weight heparin and for heterotopic ossification with indomethacin. He was able to ambulate with physical therapy on the day following surgery while remaining nonweight-bearing on the right lower extremity.

**Figure 5:**
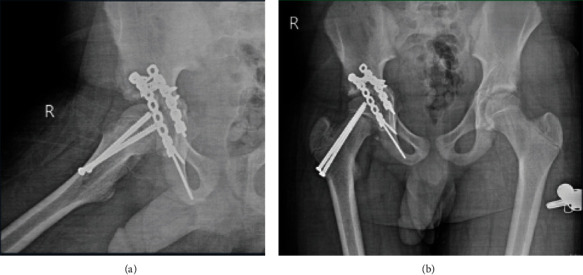
(a) AP and (b) lateral plain radiographs taken two months after fixation demonstrating the maintenance of reduction and fixation and no evidence of femoral head collapse.

**Figure 6:**
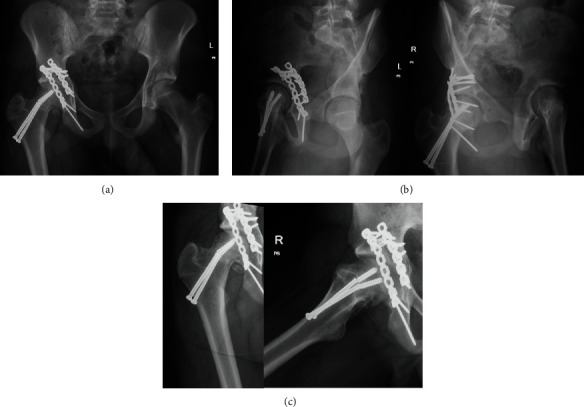
(a) AP and (b) Judet pelvis radiographs and (c) AP and lateral right hip radiographs taken 22 months after the fixation demonstrating interval healing of the right acetabulum and proximal femur, maintenance of joint space and concentricity, and no evidence of avascular necrosis. The two femoral neck screws have been broken since prior radiographic evaluation, and there is a varus malunion of the right proximal femoral physeal separation.

At the two-month follow-up, he was no longer following weight-bearing restrictions and he and his mother reported no pain or functional deficit. Radiographs (Figure 5) revealed maintenance of fixation with concentric reduction of the right femoral head and maintenance of reduction of the displaced capital femoral epiphysis. In an eighteen-month follow-up visit conducted via telephone due to coronavirus pandemic restrictions, the patient's mother reported he had no mobility restrictions and only occasional right hip pain. At an in-person follow-up visit 22 months postoperatively, the patient reported one day of groin pain approximately 13 months postoperatively and no other symptoms. On examination, he maintained a well-compensated posture in sagittal and coronal planes, ambulated with an unremarkable gait, and demonstrated grade 5/5 strength in all lower extremity muscle groups. His right hip range of motion was 0-140° flexion, 45° external rotation, and 30° internal rotation without discomfort. He lacked 5° external and internal rotation compared to the left lower extremity. Radiographs (Figure 6) revealed a healed right acetabulum and right proximal femur with interval physeal closure, preserved joint space, concentrically reduced spherical femoral head, and no evidence of avascular necrosis, confirmed upon review by our institutional board-certified musculoskeletal radiologist. Both 6.5 mm partially threaded screws in the femoral neck were broken at the base of the threads, and the proximal femoral physeal separation had healed with a slight varus malunion. The patient had resumed all activities including sporting activities such as playing basketball. He will return for reevaluation at 36 months postoperatively.

## 3. Discussion

Our report demonstrates that urgent reduction and pinning of an acute Delbet Ib displaced capital femoral epiphysis with concurrent reduction of the displaced hip joint, closely followed by osteosynthesis of the acetabulum, is a feasible treatment option for this uncommon traumatic injury pattern if referred in a timely fashion to a trauma center with an available specialist or specialists adept at caring for those injuries.

Van Nortwick et al. performed a Ganz osteotomy to repair a posterior transphyseal SH III hip fracture-dislocation in a thirteen-year-old male; he had no osteonecrosis or functional limitations at two-year follow-up [11]. Mehlman et al. found that delaying reduction over six hours increased the risk of AVN twenty-fold, and the only factor correlating with development of AVN was length of time between injury and relocation [12].

Shaath et al. reported a case of a thirteen-year-old female who sustained a transphyseal SH I femoral neck fracture and posterior hip dislocation with ipsilateral transverse-posterior wall acetabular fracture; intraoperatively, several incarcerated articular fragments were removed from the hip joint and she underwent open reduction and internal fixation of the acetabulum and femoral neck [13]. After the thirteen-year follow-up, she showed no signs of AVN and had an excellent outcome [13].

These previous similar cases and our case demonstrate that timely surgical intervention with appropriate subspecialist consultation can provide excellent outcomes in complex pelvic and acetabular traumatic injuries. In particular, this case demonstrates that staged surgical intervention can lead to excellent long-term outcomes after a traumatic acetabular injury.

Limitations of this case report include the inability to obtain close follow-up due to pandemic-related outpatient visit restrictions. However, this patient's case shows that pediatric patients can achieve good long-term results with return to all activities.

## Figures and Tables

**Table 1:** Delbet and Colonna classification of capital femoral epiphyseal injuries, after Colonna, 1928–1929 [5, 14].

I	Transepiphyseal
II	Transcervical
III	Cervicotrochanteric
IV	Pertrochanteric

## Data Availability

Please contact the corresponding author for copies of computed tomography and radiographs associated with this case report.
